# Factors associated with serious outcomes of pneumonia among children in a birth cohort in South Africa

**DOI:** 10.1371/journal.pone.0255790

**Published:** 2021-08-13

**Authors:** David M. Le Roux, Mark P. Nicol, Aneesa Vanker, Polite M. Nduru, Heather J. Zar

**Affiliations:** 1 Department of Paediatrics and Child Health, Red Cross War Memorial Children’s Hospital and SA-MRC Unit on Child and Adolescent Health, University of Cape Town, Cape Town, South Africa; 2 Department of Paediatrics, New Somerset Hospital, Cape Town, South Africa; 3 Division of Medical Microbiology, University of Cape Town and National Health Laboratory Service, Cape Town, South Africa; 4 Division of Infection and Immunity, School of Biomedical Sciences, University of Western Australia, Perth, Australia; University of Bern, SWITZERLAND

## Abstract

**Background:**

Child hospitalization for pneumonia remains common, and pneumonia is a major cause of child mortality. Early identification of clinical factors associated with serious outcomes may help target risk-mitigation strategies.

**Methods:**

Pneumonia cases occurring in the Drakenstein Child Health Study, a prospective birth cohort outside Cape Town, South Africa were analysed, and factors associated with serious outcomes of pneumonia were identified. Pregnant women were enrolled antenatally, followed through pregnancy, and mother-child pairs from birth to 2 years. Active surveillance for pneumonia was done. Children hospitalized with pneumonia had chest radiography and blood drawn for inflammatory markers; course, outcome and duration of hospitalization were investigated. Serious outcomes were defined as in-hospital mortality or admission to intensive care unit (ICU). Prolonged hospitalization was also explored as a proxy for severity. Features associated with serious outcomes or prolonged hospitalization were analysed using modified Poisson regression.

**Results:**

Among 1143 live born infants, there were 174 hospitalized pneumonia events in 133 children under 2 years. Three children (1.7%) died, 14 (8%) required ICU admission for respiratory support. In modified Poisson regression, age < 2 months, preterm birth, or hypoxia (oxygen saturation <92%) were significantly associated with serious outcomes. Preterm birth, low birth weight, HIV exposure, stunting, or underweight-for-age (UWFA) were associated with prolonged hospitalization. Chest radiography, elevated C reactive protein, white blood cell and neutrophil counts were not useful to predict death or ICU admission in children hospitalized with pneumonia.

**Conclusions:**

In this cohort, death from pneumonia was rare, but clinical features associated with serious outcomes and prolonged hospitalization were identified. These may help with risk stratification, to identify children who may benefit from enhanced monitoring or earlier escalation to respiratory support.

## Introduction

Global child deaths from pneumonia have decreased substantially over the last 30 years, from 1.7 million in 1990 to 800 000 in 2017 [1]. This decline reflects both decreased incidence and severity of pneumonia [2]. However, in low and middle income countries (LMIC), pneumonia remains the largest single cause of under-5 child mortality outside the neonatal period, and severe pneumonia is the most common reason for childhood death and hospitalization [3].

The World Health Organization (WHO) clinical classification of pneumonia changed in 2013 from 4 categories (no pneumonia, pneumonia, severe pneumonia or very severe pneumonia) to 3 categories (no pneumonia, pneumonia or severe pneumonia) [4]. These categories are intended to provide maximum sensitivity to differentiate children with community-acquired pneumonia who can safely be treated as outpatients with oral antibiotics from those who require referral to hospital for assessment and parenteral antibiotics. However, among those referred to hospital, the WHO case definition of “severe pneumonia” does not further identify those at high risk for death or admission to intensive care unit (ICU) [5,6], or those at risk for prolonged hospitalization. Risk stratifying children hospitalized with pneumonia to identify those at highest risk of serious outcomes can inform management, including enhanced monitoring, earlier initiation of supportive therapy or referral to a higher level of care.

A systematic review of hospitalized children identified 56 studies describing factors associated with serious outcomes of pneumonia [7]. Many of these were single-site retrospective cases series, included older children and teenagers (in whom case fatality is much lower), or were conducted before widespread use of conjugate vaccine against pneumococcus (PCV) and *Haemophilus influenzae* type b (Hib). More recently, the Pneumonia Etiology Research for Child Health (PERCH) study published a pneumonia severity score derived from 1802 HIV-uninfected children under 5 years hospitalized with severe or very severe pneumonia, identifying specific clinical features associated with in-hospital mortality [8]. However, all risk stratification scores are based on clinical signs and radiological features; and are cross-sectional studies, so analysis of the child’s early exposure history is limited by recall bias. To our knowledge, there are no pneumonia severity analyses nested within prospective birth cohorts, with detailed information about the early life determinants of disease, including factors around maternal health, birth, breastfeeding and early environmental or other household exposures. Although detailed early life and antenatal exposures may not be amenable for inclusion in pneumonia severity scores, exploring these is important in understanding which exposures place children at risk of serious outcomes.

We previously described incidence, severity and risk factors for pneumonia [9] and respiratory syncytial virus (RSV) pneumonia [10] in children under 2 years of age in the Drakenstein Child Health Study (DCHS), a birth cohort in South Africa. In this paper, we describe factors associated with pneumonia mortality or ICU admission in the first 2 years of life, and test whether the risk factors included in the PERCH severity score were associated with death or ICU admission in this cohort. In addition, we investigate factors associated with prolonged hospitalization for pneumonia in this birth cohort.

## Methods

### Study design

The DCHS, a population-based birth cohort established in 2012 in a peri-urban community outside Cape Town, South Africa, has been described in detail [11,12]. The population is of low socio-economic status (SES); unemployment and maternal HIV infection are common. Pregnant women (20–28 weeks’ gestation) were recruited and followed through pregnancy and delivery; mother-child pairs were followed through early childhood. For this study, follow-up through the first 2 years of life is included. University of Cape Town Faculty of Health Sciences Human Research Ethics Committee (HREC numbers 401/2009; 651/2013) provided ethical approval. Mothers provided written informed consent at enrolment, with annual renewal.

### Study procedures

Trained study staff administered structured interviews and performed home visits antenatally and postnatally. Indoor air particulate matter of diameter 10μm or less (PM_10_) was measured using an air sampling pump (AirChek 52; SKC, Eighty Four, PA, USA), placed in homes antenatally for 24 hours; the ambient standard was defined as 40 μg/m^3^ [13]. Diffusion tubes, placed in homes for 2 weeks, measured toluene concentration (Markes thermal desorption tubes; Llantrisant, UK), as described previously [14]. Maternal smoking was measured by urine cotinine measures collected antenatally and at delivery; the higher measurement was used to assign smoking status, as described previously (non smoker/passive smoker <500ng/ml; active smoker > 500 ng/ml) [14].

Pneumonia surveillance was performed using active case finding: mothers were interviewed frequently (every 2 weeks in the first year, every 3 months in the second year) about respiratory symptoms (cough, fast breathing, difficulty breathing). Thus it was possible to retrospectively identify pneumonia events occurring at other facilities or outside the area; where possible, details of these pneumonia events were obtained from clinical records of other facilities [15]. Birth cohort participants presenting to clinics or primary care providers with cough, tachypnoea or difficulty breathing were referred to the research study for assessment in real time. Trained research staff confirmed tachypnoea and clinical signs. Pneumonia was diagnosed according to revised WHO guidelines when a child had cough or difficulty breathing, with either lower chest wall indrawing (LCWI) or age-appropriate tachypnoea (≥ 50 breaths per minute if 2–12 months, ≥40 breaths per minute if ≥12 months). Severe pneumonia was diagnosed if children were under 2 months old; or at any age, with a general danger sign (cyanosis, decreased level of consciousness, inability to feed, vomiting everything, seizures) [4]. In children with wheezing, pneumonia was not diagnosed if tachypnoea and LCWI resolved following a trial of inhaled bronchodilator (5 puffs of salbutamol via a metered dose inhaler with mask/spacer). Only community-acquired cases were included. Nosocomial pneumonia (new onset respiratory symptoms occurring after hospital admission) and congenital pneumonias (tachypnoea present on the first day of life) were excluded. Pneumonia treatment and decision to admit were at the discretion of treating doctors, not study staff. Indications for hospitalization included hypoxia (oxygen saturation <92% in room air) or inability to take oral medication. Children with severe pneumonia were admitted at Paarl hospital, the single regional hospital; children requiring intensive care unit (ICU) were referred to the affiliated unit 47km away. Children were transferred to ICU for ventilatory support if they remained hypoxic (oxygen saturation <92%) despite oxygen supplementation via nasal prongs or face mask; or for inotropic support if shock was refractory to intravenous fluid therapy.

Age at pneumonia was categorized as under 2 months, 2 to 12 months or over 12 months [4]. Within each age group, duration of hospitalization was calculated; duration >90^th^ centile for age group was considered “prolonged hospitalization”. Events occurring 14 days following pneumonia were considered new-onset pneumonia. All children had anthropometry performed; stunting was defined as length-for-age <-2 Z score; and wasting as weight-for-length <-2 Z score on WHO growth charts. Severe tachypnoea or severe tachycardia were defined as respiratory rate or heart rate >99^th^ centile of normal for age [16].

Blood was drawn for full blood count, differential and C-reactive protein (CRP). CRP was modelled both as continuous and dichotomized at ≥40mg/L, as this has previously been explored as a proxy to distinguish bacterial from viral pneumonia [17]. Chest radiograph taken at the time of hospitalization was read by 2 independent doctors trained in the WHO standardized chest radiograph reporting methodology [18]. Radiographs were classified as no consolidation, “other” consolidation, or “primary end point consolidation” (PEPC). A third reader, blinded to readings of the others, resolved discordant results. Sensitivity of CRP≥ 40mg/L to predict PEPC was calculated as “true positives” (children with PEPC and CRP≥40mg/L) divided by all those with “true disease” (all those with PEPC).

Usual care was provided at one of 2 local clinics including the national vaccination schedule: Bacille Calmette Guerin (BCG) at birth; 13 valent pneumococcal conjugate vaccine (PCV) at 6, 14 weeks and 9 months; hexavalent vaccine (diphtheria, tetanus, acellular pertussis, hepatitis B, polio and Hib at 6, 10, 14 weeks and 18 months); and measles vaccine at 9 and 18 months. HIV-exposed infants were tested with HIV-DNA PCR (Cobas Ampliprep/Cobas Taqman HIV-1, Roche Diagnostics Systems, Inc., Branchburg, NJ) between 6 and 10 weeks with repeated testing during hospitalization. Testing was repeated with age-appropriate tests at 9 and 18 months and after breastfeeding cessation according to local guidelines [19]. Co-trimoxazole prophylaxis was provided to all HIV-exposed infants from 6 weeks until breastfeeding cessation.

### Statistical analysis

Continuous data were compared with median (inter-quartile range, IQR) and Wilcoxon rank-sum test. Factors associated with death/ICU admission or with prolonged hospitalization were analysed with prevalence ratios from modified Poisson regression with robust variance estimation; results of regression are presented as risk ratios and 95% confidence intervals (CI), as these are equivalent to prevalence ratios [20,21]. Robust standard errors were calculated to account for clusters within individuals. Data were analysed using Stata version 11 (College Station, Texas, USA).

## Results

From March 2012 to March 2015, 1137 pregnant women were enrolled; there were 1143 live births. Risk factors for and incidence of pneumonia in the cohort have been described [9,10]. In the first 2 years of life there were 174 episodes of hospitalized pneumonia. These 174 hospitalizations occurred in 133 children: 82/133 (62%) males; 98/133 (74%) were HIV unexposed, 34/133 (26%) were HIV exposed uninfected (HEU), 1 child was HIV infected. Median age at hospitalization was 4 months (IQR 1.8–11.0), Table 1. Among children hospitalized with pneumonia, maternal smoking (67/160, 43%), indoor air pollution (elevated levels of particulate matter, 63/103, 61%) and stunting (67/173, 39%) were common. Preterm birth (52/174, 30%) and UWFA (50/174, 29%) were less common. Severe tachycardia, severe tachypnoea, lower chest wall indrawing (LCWI) or wheezing were common; 56/171 (33%) were hypoxic. Sixteen children (9%) displayed at least 1 WHO danger sign, of which inability to feed (11 cases, 6%) was the most common. Other danger signs were rarely observed, Table 1. Pyrexia >38°C axillary temp was more common in children > 12 months than in younger children, Table 1.

**Table 1:** Characteristics of hospitalized pneumonia episodes.

	Age < 2 months (n = 47)	Age 2–12 months (n = 91)	Age >12–24 months (n = 36)
**Birth characteristics**			
Male, n(%)	31 (65%)	60 (66%)	25 (69%)
Preterm, n(%)	14 (30%)	31 (34%)	7 (19%)
Low birth weight, n(%)	9 (19%)	30 (33%)	10 (28%)
HIV unexposed, n(%)	35 (74%)	61 (67%)	29 (81%)
HIV exposed, uninfected, n(%)	12 (26%)	27 (30%)	7 (19%)
HIV infected, n(%)		3 (3%)	
**Exposures**			
Maternal education, n(%)			
Primary only	4 (9%)	11 (12%)	1 (3%)
Some secondary	26 (55%)	52 (57%)	18 (50%)
Completed secondary	17 (36%)	28 (31%)	17 (47%)
Maternal smoking^1^, n(%) (n = 156)	(n = 40)	(n = 82)	(n = 34)
Non smoker/passive smoker	22 (55%)	41 (50%)	26 (76%)
Active smoker	18 (45%)	41 (50%)	8 (24%)
Household crowding, n(%)	23 (49%)	28 (31%)	12 (33%)
6 or more in house			
Ever breastfed, n(%)	43 (91%)	80 (88%)	29 (81%)
Indoor air pollution: PM_10_ ^2^, n(%) (n = 103)	(n = 21)	(n = 56)	(n = 26)
Below ambient standard	10 (48%)	16 (29%)	14 (54%)
Above ambient standard	11 (52%)	40 (71%)	12 (46%)
Toluene, n(%) (n = 105)	(n = 22)	(n = 56)	(n = 27)
Below ambient standard	19 (86%)	52 (93%)	23 (85%)
Above ambient standard	3 (14%)	4 (7%)	4 (15%)
Routine vaccinations (n = 160)	(n = 39)	(n = 87)	(n = 34)
All on time, n(%)	21 (54%)	44 (51%)	20 (59%)
All given but some late, n(%)	16 (41%)	42 (48%)	13 (38%)
Some doses missed, n(%)	2 (5%)	1 (1%)	1 (3%)
**WHO danger signs**			
Unable to feed	2 (4%)	6 (7%)	3 (8%)
Decreased level of consciousness, n(%)	1 (2%)	1 (1%)	2 (6%)
Vomiting everything	0	3 (3%)	2 (6%)
Convulsions	0	1 (1%)	2 (6%)
Central cyanosis	0	0	1 (3%)
Any WHO danger sign	3 (6%)	9 (10%)	4 (11%)
**Clinical features**			
Duration of illness prior to presentation, days, median (IQR)	3 (2–5)	3 (2–4)	3 (1–4)
Underweight for age^3^, n(%) n = 173	11 (23%)	32 (36%)	7 (19%)
Stunted^4^, n(%) n = 173	17 (36%)	43 (48%)	7 (19%)
Wasted^5^, n(%) n = 169	5 (11%)	8 (9%)	7 (19%)
Grunting	13 (28%)	18 (20%)	5 (14%)
Head nodding	11 (24%)	6 (7%)	1 (3%)
Cough not observed	22 (49%)	24 (27%)	12 (33%)
Pyrexia >38°C on presentation, n(%)	9 (20%)	44 (50%)	24 (67%)
Pulse rate, median (IQR)	171 (160–185)	168 (158–182)	159 (146–169)
Severe tachycardia^6^, n(%)	13 (28%)	46 (50%)	22 (61%)
Respiratory rate, median (IQR)	68 (60–72)	60 (56–67)	54 (47–64)
Severe tachypnoea^7^, n(%)	25 (53%)	34 (37%)	23 (64%)
Lower chest indrawing, n(%)	41 (87%)	86 (95%)	23 (64%)
Wheeze, n(%)	22 (47%)	55 (60%)	15 (42%)
Hypoxic (saturation <92%), n(%)	15 (33%)	30 (34%)	11 (31%)
**Chest radiograph findings, n(%) (n = 162)**	(n = 43)	(n = 85)	(n = 34)
No consolidation	15 (35%)	16 (19%)	5 (15%)
Other consolidation	13 (30%)	33 (39%)	16 (47%)
Primary end point consolidation	15 (35%)	36 (42%)	13 (38%)
**Laboratory features**			
White cell count^8^, median (IQR)	10.6 (8.9–14.2)	13.4 (10.5–16.9)	15.2 (10.6–20.7)
Neutrophil count^8^, median	3.2 (2.4–6.3)	5.3 (3.2–7.3)	7.3 (5.1–16.6)
(IQR)			
Neutrophil percentage, median (IQR)	33 (21–38)	40 (28–52)	56 (38–68)
Haemoglobin, g/dL, median (IQR)	11.4 (9.8–12.8)	10.1 (9.4–11.1)	11 (9.8–11.7)
C reactive protein, mg/L, median (IQR)	4 (2–20)	8 (3–38)	29 (13–49)
**Outcomes**			
Duration of hospitalization median (IQR)	4 (2–6)	3 (2–5)	2.5 (1–4)
Prolonged hospitalization^9^	5 (11%)	10 (11%)	4 (11%)
Died or admitted to ICU^10^, n(%)	9 (19%)	7 (8%)	0

1) Based on antenatal urine cotinine: Non smoker/passive smoker <500ng/ml; active smoker ≥ 500 ng/ml.

2) Particulate matter <10 micrometer.

3) Weight for age <-2 Z score.

4) Length for age <-2 Z score.

5) Weight for length <-2 Z score.

6) Heart rate > 99^th^ centile for age.

7) Respiratory rate > 99^th^ centile for age.

8) Cell counts: x 10^9^/ L.

9) Prolonged hospitalization: Duration ≥90^th^ centile for age category.

10) ICU: Intensive care unit.

Of the 174 hospitalized pneumonia cases, there were 3 deaths (1.7%); 14 (8%) were admitted to ICU for respiratory support: 8 required intubation and mechanical ventilation and 6 received continuous positive airway pressure (CPAP), Table 2. One child died in ICU and 2 died prior to ICU transfer.

**Table 2:** Clinical description of children with serious outcomes of pneumonia (death or ICU admission).

Sex	Birth weight, kg	Gestation, months	Age at pneumonia event, months	HIV status	Duration of illness days	Oxygen saturation, percent	WHO danger signs	Chest radiograph WHO category	CRP (mg/L)	Respiratory support	Outcome
Male	3.37	34	0.3	Exposed	(not recorded)	93	Decreased level of consciousness	No consolidation	20	IPPV	Survived
Female	2.69	36	0.8	Unexposed	2	81		Primary end point	117	IPPV	Died
Male	3.31	41	1	Exposed	3	99		Primary end point	4	CPAP	Survived
Male	2.61	39	1	Unexposed	2	93		Primary end point	51	CPAP	Survived
Male	2.46	37	1	Unexposed	1	92		Other consolidation	??	IPPV	Survived
Male	1.30	29	1.5	Unexposed	4	99		No consolidation	4	IPPV	Survived
Male	2.84	36	1.5	Unexposed	1	40		Primary end point	44	IPPV	Survived
Female	3.31	42	1.5	Unexposed	4	73	Unable to feed	Primary end point	7	CPAP	Survived
Male	2.72	40	1.5	Unexposed	2	(not done)		(not done)		(nil)	Died
Male	1.19	27	3	Unexposed	1	94		Primary end point	3	IPPV	Survived
Female	1.06	28	3	Unexposed	3	79		Primary end point		CPAP	Survived
Male	1.06	32	4	Exposed	4	86	Unable to feed	Primary end point	76	CPAP	Survived
Male	1.06	32	5	Exposed	7	85	Decreased level of consciousness Unable to feed	Primary end point	132	IPPV	Survived
Female	3.44	39	6	Unexposed	2	88		Primary end point	11	CPAP	Survived
Male	1.06	32	8	Exposed	2	90		Primary end point		IPPV	Survived
Female	3.19	38	11	Unexposed	2	79		Primary end point	9	(nil)	Died

WHO: World Health Organization.

CRP: C reactive protein.

IPPV: Intermittent positive pressure ventilation.

CPAP: Continuous positive airway pressure.

### Fatal pneumonia or ICU admission

Fatal pneumonia or ICU admission were strongly associated with younger age: 2 of the 3 deaths and 8 of the 14 ICU admissions occurred in children under 2 months. In unadjusted modified Poisson regression, age <2 months, preterm birth, and hypoxia were significantly associated with serious outcomes, Table 3. WHO danger signs of decreased level of consciousness and inability to feed, although rarely observed, were strongly associated with serious outcomes. Low birth weight, maternal smoking, UWFA, stunting, grunting, radiological consolidation (PEPC), and elevated CRP appeared to be associated with increased risk of serious outcomes, but precision for these estimates was limited. Female sex, wasting, duration of illness prior to presentation, and cough not observed were not associated with serious outcomes. Several factors were identified which, although previously associated with pneumonia incidence, were not associated with serious outcomes: male sex, household crowding, HIV exposure, incomplete vaccinations, and inadequate breastfeeding. Children exposed to significant household air pollution, measured by elevated antenatal PM_10_ concentration, had a 3-fold increased risk of a serious outcome, but precision for this estimate was limited by small sample size (OR 3.17, 95% CI 0.38–26.46).

**Table 3:** Factors associated with A) Death or admission to Intensive Care Unit (ICU); and B) Prolonged hospitalization: Unadjusted risk ratios from modified Poisson regression.

Clinical characteristics	A: Death or ICU admission (n = 16)	B: Prolonged hospitalization^1^ (n = 19)
**Birth characteristics**	Risk ratio	Risk ratio
Male	1.10 (0.40–3.03)	2.66 (0.81–8.81)
Pre-term	**3.02 (1.18–7.69)**	**3.23 (1.37–7.57)**
Low birth weight	1.98 (0.78–5.05)	**3.51 (1.50–8.22)**
HIV unexposed	1	1
HIV exposed, uninfected	0.91 (0.31–2.68)	**3.74 (1.60–8.72)**
HIV infected	(unable)	(unable)
**Exposures**		
Maternal education, n(%)		
Primary only	1	1
Some secondary	1.66 (0.23–12.22)	1.00 (0.25–4.07)
Completed secondary	1.29 (0.16–10.34)	0.65 (0.11–3.42)
Household crowding: 6 or more in house	0.59 (0.20–1.75)	0.46 (0.16–1.36)
Maternal smoking: Active smoker^2^	2.32 (0.71–7.65)	1.33 (0.45–3.95)
Never breastfed	0.46 (0.06–3.34)	**3.19 (1.35–7.54)**
PM_10_: Above ambient standard	3.17 (0.38–26.46)	1.59 (0.32–7.85)
Late or missing vaccinations	1.40 (0.31–6.24)	(unable)
**WHO danger signs**		
Unable to feed	**3.38 (1.12–10.15)**	2.74 (0.94–8.04)
Decreased level of consciousness	**6.00 (1.99–18.10)**	**4.94 (1.68–14.58)**
Vomiting everything	(unable)	(unable)
Convulsions	(unable)	3.13 (0.59–16.52)
Central cyanosis	(unable)	(unable)
Any WHO danger sign	**3.29 (1.20–9.04)**	1.85 (0.60–5.70)
**Clinical**		
Age: over 2 months	1	
Under 2 months	**3.47 (1.36–8.83)**	
Duration of illness prior to presentation, days	0.83 (0.64–1.08)	0.78 (0.58–1.04)
Underweight for age^3^	2.46 (0.97–6.21)	**5.33 (2.14–13.27)**
Stunted^4^	1.58 (0.62–4.02)	**2.71 (1.12–6.56)**
Wasted^5^	0.62 (0.08–4.55)	2.29 (0.83–6.36)
Grunting	2.50 (0.95–6.58)	1.73 (0.71–4.25)
Head nodding	1.21 (0.30–4.94)	1.00 (0.25–4.00)
Cough not observed	1.43 (0.52–3.95)	1.53 (0.64–3.68)
Pyrexia >38°C on presentation	0.30 (0.09–1.04)	0.70 (0.29–1.71)
Severe tachycardia^6^	0.69 (0.26–1.82)	1.97 (0.81–4.77)
Respiratory rate	0.98 (0.94–1.02)	0.99 (0.96–1.02)
Severe tachypnoea^7^	0.87 (0.34–2.24)	0.82 (0.34–1.93)
Lower chest indrawing	1.12 (0.27–4.64)	1.36 (0.33–5.54)
Wheeze	0.89 (0.35–2.27)	0.99 (0.42–2.32)
Hypoxic (saturation <92%)	**3.08 (1.15–8.25)**	1.85 (0.79–4.30)
**Chest radiograph findings**		
No consolidation	1	1
Other infiltrate	0.29 (0.03–3.11)	2.32 (0.27–20.12)
Primary end point consolidation	3.38 (0.80–14.31)	**7.88 (1.07–57.81)**
**Laboratory features**		
C reactive protein		
< 40mg/L	1	**1**
≥40 mg/L	2.41 (0.82–7.11)	**3.00 (1.26–7.16)**
White cell count	1.02 (0.96–1.09)	**1.05 (1.02–1.08)**
Neutrophil count	1.00 (0.91–1.10)	**1.08 (1.03–1.14)**
Neutrophil percentage	1.01 (0.97–1.05)	**1.03 (1.01–1.06)**
Haemoglobin, g/dL	1.10 (0.76–1.59)	0.84 (0.66–1.08)

1. Prolonged hospitalization: Duration of hospitalization >90^th^ centile, by age category.

2. Based on antenatal urine cotinine: Non smoker/passive smoker <500ng/ml; active smoker ≥ 500 ng/m.

3. Weight for age <-2 Z score.

4. Length for age <-2 Z score.

5. Weight for length <-2 score.

6. Heart rate >99^th^ centile for age.

7. Respiratory rate >99^th^ centile for age.

### Duration of hospitalization

Median duration of hospitalization was 3 days (IQR 2–5); children under 2 months had longer duration of hospitalization compared to children over 12 months, 4 days (IQR 2–6) vs 2.5 days (IQR 1–4), p = 0.04, Table 1. HEU children had longer hospitalization (median 4 days, IQR 2–8) compared to HIV unexposed children (median 3 days, IQR 2–4, p = 0.004). Prolonged hospitalization was associated preterm birth, low birth weight, HIV-exposure, never breastfeeding, decreased level of consciousness, UWFA and stunting. Hypoxia, lower chest indrawing and other WHO danger signs were not associated with increased risk of prolonged hospitalization, Table 3. PEPC on chest radiograph, elevated CRP, total white cell and neutrophil counts were also associated with prolonged hospitalization.

### Chest radiography and C-reactive protein

Of 174 pneumonia hospitalizations, 162 (93%) had chest radiographs reported. Overall, 64 (40%) had “primary end point consolidation” (PEPC) and 62 (38%) had “other consolidation”. PEPC was equally common in all age categories, Table 1. CRP result was available for 140/174 (80%) of hospitalized pneumonia events; median CRP was higher in children older than 12 months compared to those aged 2–12 months (p = 0.002) or children aged < 2months (p<0.0001), Table 1. Children with PEPC tended to have higher CRP levels (median 19 mg/L, IQR 5–51) than children with “other consolidation” (median 10mg/L, IQR 3–39) or “no consolidation” (median 4 mg/L, IQR 3–18); but only 18/51 (35%) of children with PEPC had CRP>40mg/L. CRP cut-off of 40mg/L did not predict death or ICU admission (sensitivity 42%) or prolonged hospitalization (sensitivity 47%).

## Discussion

In this well-vaccinated birth cohort with negligible HIV infection and reasonable nutritional status, 1.7% of hospitalized pneumonia cases died and 8% required ICU admission. Good access to care and referral may have also contributed to low pneumonia mortality. Young age < 2 months, hypoxia, or preterm birth were strongly associated with death or ICU admission; while low birth weight, stunting, UWFA, HIV exposure, and WHO-defined radiographic consolidation were strongly associated with prolonged hospitalization.

Hypoxia has been considered the most important feature of pneumonia severity [5,22–24]. However, in our cohort, peripheral oxygen saturation <92% was a reason to consider hospitalization, and was very common, occurring in 33% of hospitalized cases. Hypoxia was associated with serious outcomes, but was so common in this cohort that it did not have good discriminatory value among hospitalized children to identify those who require intensive monitoring or additional respiratory support. There are no reliable clinical signs to predict hypoxia in children [25], which makes pulse oximetry screening and oxygen supply essential for all facilities treating children [26].

Preterm delivery confers multiple risks for childhood morbidity, including less transplacental antibody transfer, anaemia, suboptimal breastfeeding, delayed/missing vaccinations, and slower growth [27,28]. Preterm birth is useful as a risk stratification marker where gestational age is accurately known; front-line health care workers can quickly identify at-risk children from patient-held birth records, and prioritise them for urgent assessment and pulse oximetry testing.

The original definition of WHO standardized radiographic consolidation was intended as a proxy marker of bacterial pneumonia for use in vaccine studies [18], and was strongly associated with mortality [29]. However, there is increasing evidence that much severe pneumonia post PCV and Hib is viral: 33% of pneumonia hospitalizations in this cohort were associated with RSV [10], and in the PERCH study, viruses (especially RSV) were predominant causes of hospitalized pneumonia among children with positive chest radiology [30]. In this well-vaccinated cohort, neither radiological consolidation nor elevated CRP were significantly associated with death/ICU admission. Furthermore, although PEPC was associated with higher median CRP, there was no CRP threshold that strongly predicted PEPC. It is increasingly accepted that CRP is a poor marker of bacterial infection [31,32]. This is consistent with the epidemiologic change that has occurred since WHO radiographic scoring was developed [33]; so PEPC or elevated CRP should no longer be considered a proxy for bacterial infection. Since neither radiological consolidation nor elevated CRP strongly predicted death or ICU admission, and are not widely available in LMIC, they could be excluded from severity scores in favour of more easily-obtainable clinical features.

A recent systematic review of pneumonia risk stratification scores for children in low-resource settings also described hypoxia, LCWI and decreased level of consciousness as strongly predictive for mortality, but that wheeze was protective [7]. A pneumonia risk stratification prediction model developed and validated in a high-income country reported retractions were associated with severe outcomes, but did not report prevalence of wheeze [6]. In our analysis, both wheezing and LCWI were common, but not associated with serious outcomes. This is consistent with evidence that led to WHO reclassification of LCWI as a sign of pneumonia for ambulatory therapy, not a marker of severe pneumonia requiring parenteral therapy [4]; in these young children, where oxygen saturation monitoring was available, LCWI indrawing did not add value in identifying those at risk of severe outcomes [34].

The PERCH severity score identified infants < 1 year, female sex, decreased level of consciousness, fast breathing without observed cough, grunting, hypoxaemia (peripheral oxygen saturation less than 90%), duration of illness more than 3 days and severe wasting as risk factors for mortality [8]. The score had a moderate overall predictive accuracy (C statistic 0.76% in a validation data set), but lower predictive accuracy in some age groups (eg age 6–11 months, where mortality was highest) and some sites, including South Africa, where mortality was lowest. The authors recommended that the PERCH score should be validated with other datasets. In our cohort, some of the PERCH predictors were confirmed: young age and hypoxia were strongly associated with serious outcomes. Decreased level of consciousness was infrequently identified, limiting its practical usefulness as a predictor of serious outcomes. Other PERCH predictors performed poorly in our study: female sex, grunting, cough not observed, and longer duration of illness were not associated with serious outcomes; no wasted child died or required ICU admission. Furthermore, the PERCH tool may be impractical for frontline clinicians in LMIC, as it requires careful history from the mother or caregiver, and accurate plotting of weight-for-length, which is often poorly recorded in critically ill children.

A strength of this study is the availability of detailed, longitudinal exposure history prior to pneumonia. Certain environmental factors, measured before the pneumonia event (antenatal maternal smoking, household crowding) were associated with hospitalization for pneumonia [9], but were not associated with serious outcomes. This suggests that while maternal smoking and household crowding were significant risk factors in the causal pathway for developing pneumonia, they may not be associated with severity; or may have been too common in this cohort to predict serious outcomes with adequate discrimination. Although elevated household PM_10_ level was not significantly associated with risk of serious outcomes, precision for this estimate may have been limited by sample size. Indoor air pollution plays a critical role in child lung health [14]; this could be investigated as modifiable factor in adequately-powered prospective interventions.

Prolonged hospitalization may be used as a valid measure of pneumonia severity [35]; however, in children with complex co-morbidities, duration of hospitalization may reflect the underlying medical or social problems, and not the severity of the respiratory infection. Preterm birth, HIV exposure, stunting, underweight-for-age, and elevated CRP were significantly associated with prolonged hospitalization. We cannot attribute causality to these associated factors, as reasons for prolonged hospitalization may have been due to the severity of the respiratory infection, but also may have been due to other associated illnesses, nutritional rehabilitation or addressing the social concerns or feeding practices. We previously showed that HEU children have increased incidence of pneumonia in the first 6 months of life [9]; in this analysis we show that they are not at greater risk of serious outcomes, although they are at increased risk of prolonged hospitalization.

Limitations of this study include inadequate power to detect an association, as both death and ICU admission were such rare events; even when they are combined as a composite endpoint of “death or ICU admission”, power is limited. Furthermore, use of a composite endpoint requires caution, as some clinical features may reflect the bias of the treating clinician, eg clinicians could be more likely to refer a low birth weight child to ICU, even if the severity of the disease did not require respiratory support. However, this is unlikely as the ICU in the regional referral hospital was 47km away, is a very limited resource, and all children admitted to ICU received invasive or non-invasive ventilation. Some authors consider non-ventilated ICU admissions “moderate” severity, vs death/ventilation (“severe”) [5]. As we had few events with the composite outcome, we did not analyse “moderate” outcomes separately, but considered all ICU admissions as serious. Prediction models should be derived from one dataset and then applied to a different dataset, or validated with internal bootstrap [8,36,37]. With this small dataset, we were unable to derive a true prediction model; but results compare favourably with predictors from other local and international models. Pneumonia surveillance was strong and consistent throughout the study period; follow up of individuals and overall cohort retention was good, so missed pneumonia hospitalizations or deaths are unlikely. Other missing data, particularly CRP and white cell counts, would not have made substantial impact on outcomes, as most cases with serious outcomes had all blood results available.

In conclusion, we observed low mortality from pneumonia in this cohort. Most of the PERCH severity predictors performed poorly in this cohort. Clinical factors associated with death or ICU admission included age under 2 months, preterm birth or hypoxia. These clinical measures are easily obtained, do not require radiography, blood tests or interpretation of growth charts, and further underscore the urgent need for more widespread availability of pulse oximeters and oxygen in LMICs. Presence of these risk factors should alert clinicians to identify children who may require additional monitoring or early escalation of care; clinicians should be encouraged to engage in appropriate risk-stratification to identify at-risk children.
